# Study on mechanical properties and fracture surface characteristics of argillaceous sandstone under different stress paths

**DOI:** 10.1038/s41598-022-20433-y

**Published:** 2022-09-27

**Authors:** Tianzhu Huang, Lehua Wang, Jianlin Li, Bingyi Zhang, Xiaoping Wang, Xiaoliang Xu

**Affiliations:** 1 Key Laboratory of Geological Hazards on Three Gorges Reservoir Area, Ministry of Education, China Three Gorges University, Yichang, 443002 Hubei China; 2 College of Civil Engineering and Architecture, China Three Gorges University, Yichang, 443002 Hubei China

**Keywords:** Civil engineering, Geophysics

## Abstract

To study the differences in mechanical properties and failure characteristics of underground chambers surrounding rock under different stress conditions, triaxial loading and unloading tests were carried out on argillaceous sandstone. The three-dimensional topography parameters of the fracture surface were obtained by using high-precision three-dimensional topography scanning technology, including six height characteristic parameters and two texture parameters. Compared with the triaxial loading test, the strength, peak strain and residual strength of argillaceous sandstone with the same confining pressure under unloading conditions all decrease, and the stress–strain curve changes from ductility to brittleness. The Mogi–Coulomb strength criterion can better describe the strength properties of argillaceous sandstones than the Mohr–Coulomb and Drucker–Prager strength criteria. Under the unloading condition, the cohesion *c* decreased by 30.87% and the internal friction angle *φ* increased by 30.87% compared with the loading condition. The tensile cracks perpendicular to the unloading direction is formed during unloading, resulting in large roughness, dispersion and fluctuation of fracture surface.

## Introduction

The stress of the underground cavern surrounding rock will be readjusted under the action of excavation and unloading, which is usually accompanied by the cracking, rockburst and other damages of the surrounding rock^1,2^. Surrounding rock failure and instability are essentially the evolution process of rock mass meso-damage to macro-cracks in the process of stress adjustment. Moreover, the state and change of the stress of the rock mass will affect its mechanical properties^3,4^. Therefore, in order to accurately analyze the stability of the surrounding rock of the underground cavern, it is necessary to compare the macroscopic and mesoscopic deformation and failure processes of the rock mass under loading and unloading.

Triaxial unloading test studies of rocks under different unloading paths show that unloading will cause strong volume failure of the rock along the unloading direction, and rock under unloading are more easily damaged than under loading^5–7^. In addition, triaxial unloading experiments under different initial damages, unloading rates and stress levels were carried out, and the influence of various factors on the rock were analyzed^8–10^. Various rock strength and failure criteria can reflect the strength characteristics of unloading rocks based on unloading tests, among which are the Mohr–Coulomb criterion^11^, Hoek–Brown criterion^12^ and Mogi–Coulomb criterion^13^ that are often used. Wu et al.^14^ showed that the Mohr Coulomb failure criterion cannot accurately describe the strength characteristics of rock in unloaded state. Yang et al.^15^ proposed that the Hoek Brown criterion is better than the Mohr Coulomb criterion to describe the strength properties. Lu et al.^16^ pointed out that the Mogi-Coulomb failure criterion can better reflect the strength characteristics of granite under stress unloading conditions. However, which failure criterion is more suitable to reflect the unloading strength characteristics of rock remains to be discussed.

For the study of the failure mechanism of unloaded rock mass, the macro-level analysis of the basic mechanical behavior and failure characteristics is no longer sufficient. Li et al.^17^ studied the rock failure mechanism based on the law of energy storage, dispersion and release during rock failure. In addition, Qiu et al.^18^ showed that the failure of rocks under various stress paths is related to the development and propagation of cracks. Therefore, understanding the development process of microcracks is helpful to the understanding of rock failure mechanism. Cong et al.^19^ studied Macro- and Microfracture Mechanisms of brittle rocks with different unloading stress levels using acoustic emission technology and particle flow numerical simulation methods. Zhou et al.^20,21^ studied the fracture characteristics of rock samples at high temperature using industrial cameras and digital image correlation techniques, and also studied the crack propagation characteristics of single-flawed tunnel specimens under dynamic loading combined with experiments and numerical simulations. Zhao et al.^22^ combined acoustic emission and high-speed camera technology to study the fracture development process of Beishan granite at different unloading rates. Zhou et al.^23^ combined the CT technology to obtain the whole process of rock from the compaction of micro-cavities to the occurrence, development, penetration and destruction of micro-cracks. However, due to the limitation of experimental techniques, the whole process of monitoring the development of micro-fractures cannot be widely used. The meso-geometric characteristics of fracture surfaces are determined by the development of cracks, which significantly affect the stability of rock sliding along the shear plane^24,25^. The development of microcracks can be well studied by comparing the fine microstructure of the fracture before and after the rock test by using SEM, CT, and topography scanner^26,27^.

Therefore, this paper takes argillaceous sandstone as the research object, and carries out triaxial loading and triaxial constant axial pressure unloading confining pressure tests to study the mechanical properties of argillaceous sandstone under different stress paths. Three-dimensional morphology scanning technology is used to compare and analyze the change law of argillaceous sandstone fracture surface morphological characteristic parameters under loading and unloading conditions. Comprehensive shear strength parameters, failure characteristics and fracture surface morphology characteristics to analyze the failure mechanism of argillaceous sandstone. The results provide guidance for the prevention and control of disasters related to the underground chambers rock surrounding.

## Experimental program design

### Preparation of samples

**Figure 1:**
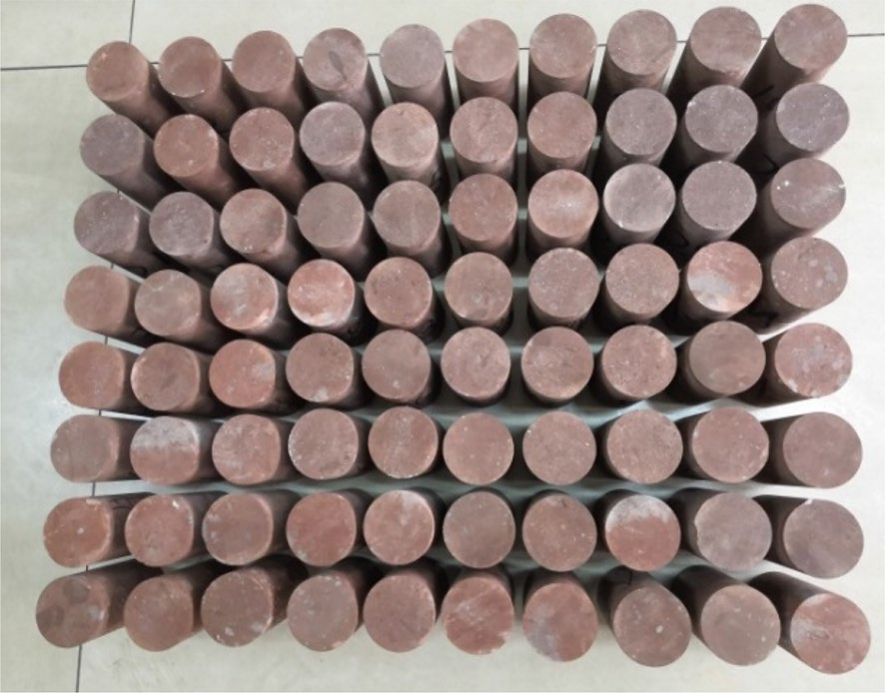
Argillaceous sandstone samples.

The samples are argillaceous sandstones selected on site. According to the rock sample preparation standard^28^ the standard cylindrical sample (Φ 50mm × 100 mm) was produced by drilling, cutting and polishing in the laboratory, as shown in Fig. 1. Select the test with similar wave speed, mass and density for testing. The wave speed of the sample at normal temperature is 2900–2950 m/s, and the density is 2.63 g/cm^3^. The chemical composition and mineral composition of the rock are detected and identified. The main chemical composition is SiO_2_, CaO, Al_2_O_3_, Fe_2_O_3_ and MgO, the contents are 60.54%, 10.66%, 9.11%, 3.46% and 1.83%, respectively. In the mineral composition, the clastics mainly include quartz (42%), calcite (14%), mica (9%) and rock fragments (8%), and the cement mainly includes clay minerals (18%) and calcite (10%).

### Test methodology

**Figure 2:**
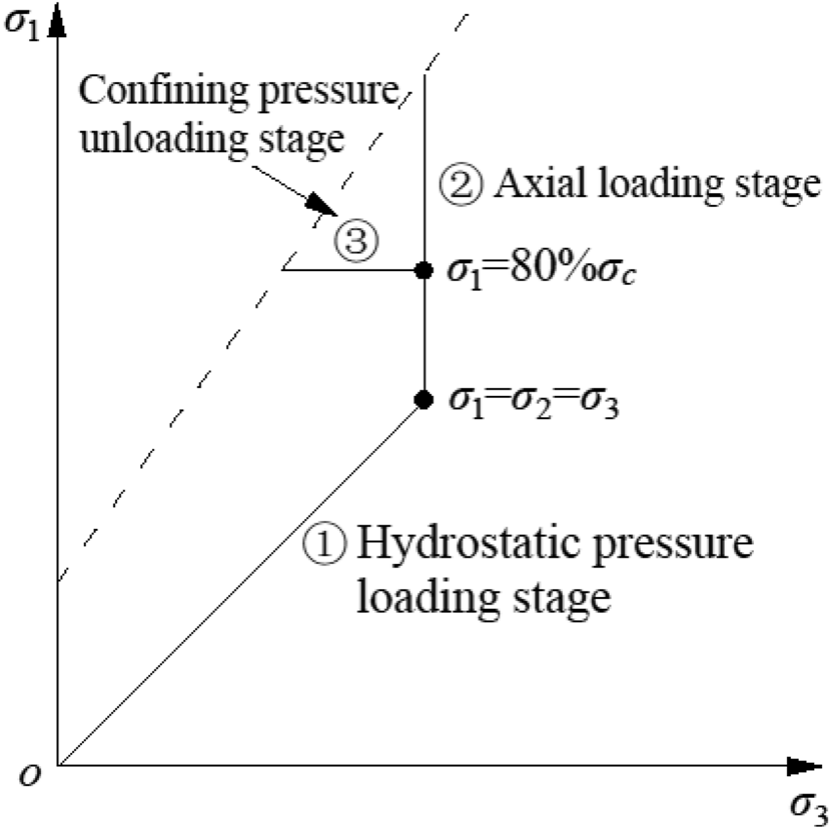
Triaxial loading and unloading test stress paths.

The selected argillaceous sandstone is the surrounding rock of the tunnel, the maximum burial depth is about 500 m, and the maximum in-situ stress level is about 15 MPa, so the confining pressure of the test is taken as 5 MPa, 10 MPa and 15 MPa respectively. The tests are all carried out on the RMT-150C rock triaxial testing machine, and the test stress paths is shown in Fig. 2.

The triaxial loading test: ① The hydrostatic pressure loading stage, the axial and confining pressure were simultaneously applied to the test design values (5 MPa, 10 MPa, 15 MPa). ② The axial loading stage, the confining pressure is fixed and the axial pressure is loaded until the rock sample is completely destroyed. The triaxial unloading test: ① The hydrostatic pressure loading stage is consistent with the stress path of the triaxial loading test. ② The axial loading stage, the axial pressure was applied to 80% *σ*_*c*_, and *σ*_*c*_ is the triaxial peak strength under different confining pressures. ③ The confining pressure unloading stage, the axial pressure is fixed, and the confining pressure is unloaded until sample is completely destroyed.

**Figure 3:**
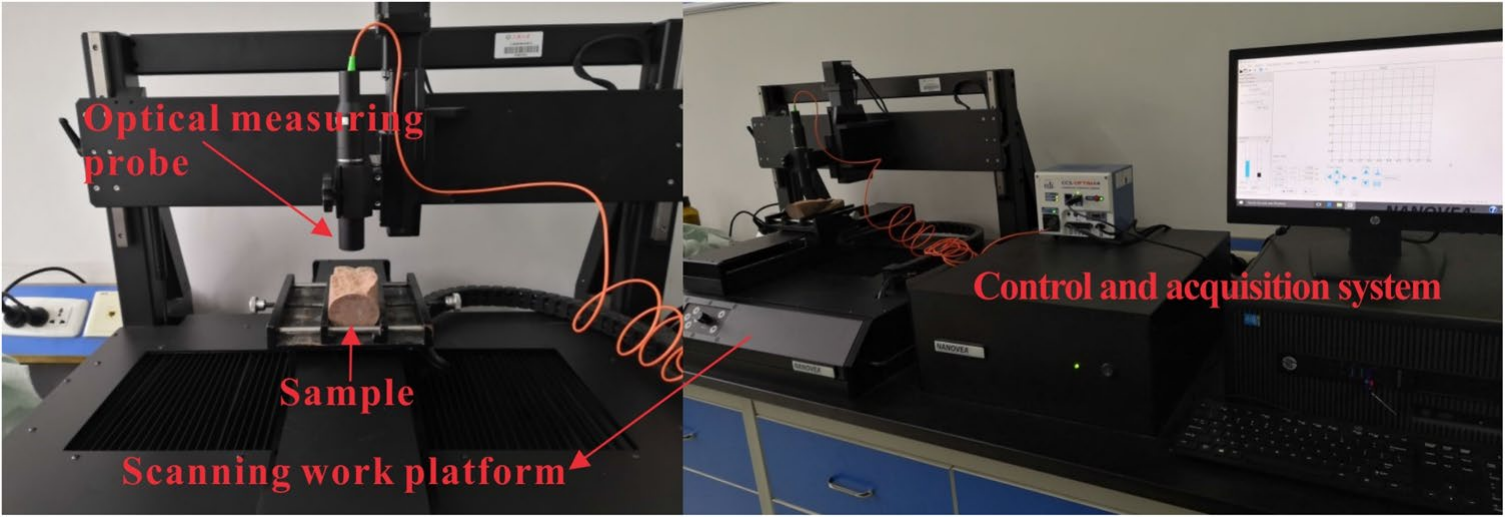
ST500 three-dimensional non-contact surface profiler.

After taking out the damaged sample, the fracture surface was scanned with the ST500 three-dimensional non-contact topography scanner (Fig. 3). The scanning direction of all fracture surfaces is the same, the scanning range is a rectangle of 50 mm × 100 mm, and the sampling interval during scanning is 300 μm.

## Results and analysis

### Stress–strain curves

**Figure 4:**
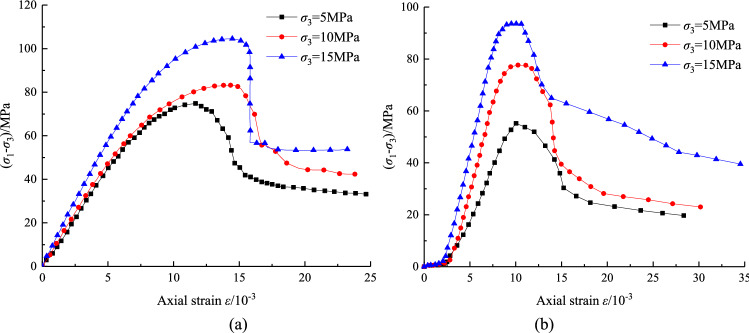
Stress–strain curves : (**a**) loading, (**b**) unloading.

The stress–strain curves of the triaxial loading and unloading tests are shown in Fig. 4, and the test results are shown in Table 1. It can be seen that the rocks under different stress paths show obvious elastoplastic characteristics, and the curves changes from a linear relationship to a nonlinear relationship before reaching the peak strength. Compared with the loading condition, the strain softening tendency is weakened and the brittleness characteristics are more obvious of the rock under the unloading condition, and the peak strength and peak strain of the sample are lower.

**Table 1:** Triaxial loading and unloading test results.

Test type	Confining pressure *σ*_3_ (MPa)	Confining pressure at failure *σ′*_3_ (MPa)	Peak deviator stress *σ*_1_ *−* *σ*_3_, *σ*_1_ *−* *σ′*_3_ (MPa)	Residual stress *σ*_1r_ (MPa)	Peak strain *ε*_*p*_ (10^–3^)
Triaxial loading test	5	–	69.44	34.18	11.64
	10	–	89.13	42.89	14.05
	15	–	105.59	53.39	14.47
Triaxial unloading test	5	1.2	54.8	19.69	10.05
	10	3.0	76.0	22.99	10.24
	15	7.5	87.5	39.54	10.59

### Failure characteristics

**Figure 5:**
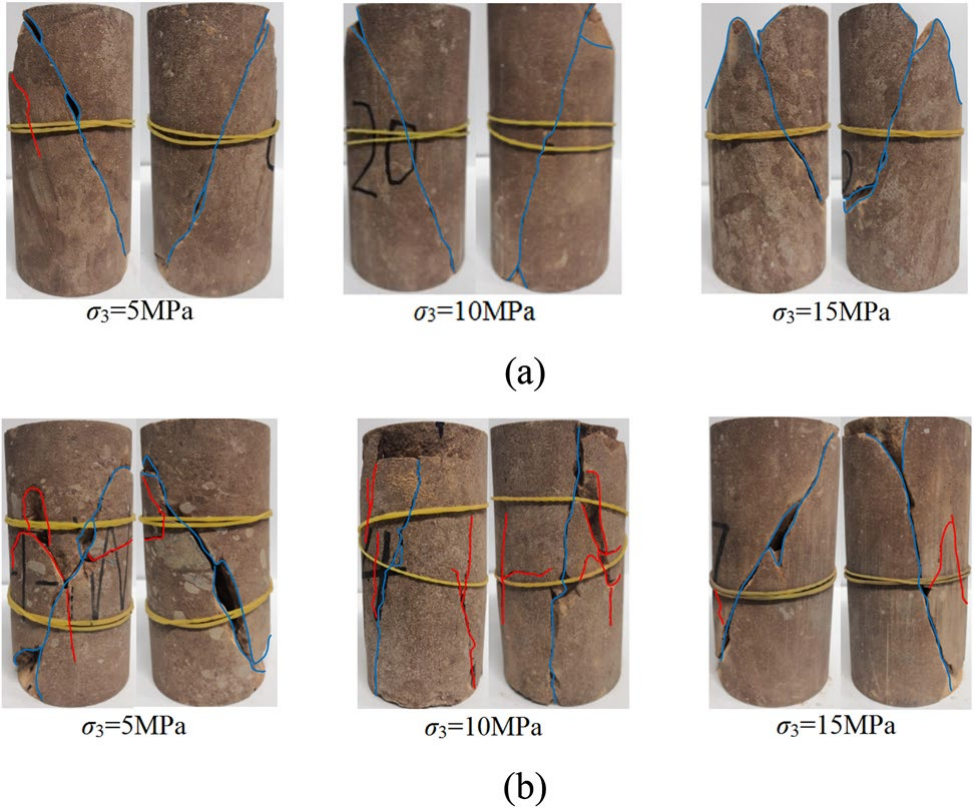
Failure characteristics of argillaceous sandstone: (**a**) triaxial loading test, (**b**) triaxial unloading test.

Figure 5 show the failure characteristics of argillaceous sandstone under different stress paths, respectively, and both show shear failure characteristics (blue curve is the shear failure surface). The fracture surface during loading is narrow and the degree of agreement is high, indicating that the development and penetration of compression-shear fractures caused argillaceous sandstone to undergo compression-shear failure. The fracture surface during unloading is wide, and powder and fragments are dropped. A number of secondary tensile cracks extend near the main shear plane, indicating that the rock deforms in the unloading direction and the rock sample undergoes tensile and shear failure. When the confining pressure is relieved, the high axial stress level causes the rapid development and propagation of microcracks. The high confining pressure will slow down the development of secondary cracks such as tensile cracks, resulting in the reduction of secondary cracks as the unloading confining pressure increases.

### Shear strength parameters analysis

The Mohr–Coulomb strength criterion believes that the sum of the cohesive force of the rock and the friction force generated by the normal stress on the shear surface is the shear strength of the rock. The cohesive force *c* and the internal friction angle *φ* can be obtained through the stress state when the rock undergoes shear failure. The expression is^29^:

1 $$ \sigma_{1} { = }A\sigma_{3} + B $$

where *σ*_1_ and *σ*_3_ are the maximum and minimum principal stresses when the rock failure, respectively, $A = \frac{1 + \sin \varphi }{{1 - \sin \varphi }}$, $B = \frac{2c\cos \varphi }{{1 - \sin \varphi }}$.

Using Eq. (1) to perform regression analysis on the data in Table 1, the parameters *A* and *B* can be obtained, and *c* and *φ* can be determined by:

2 $$ \varphi = \arcsin \frac{A - 1}{{A + 1}} $$

3 $$ c = \frac{{B\left( {1 - \sin \varphi } \right)}}{2\cos \varphi } $$

The Mohr–Coulomb strength criterion does not consider the influence of the intermediate principal stress. Through a large number of rock triaxial compression tests, Mogi^30^ believes that the influence of intermediate principal stress on rock strength cannot be ignored, and proposed the octahedral strength criterion:

4 $$ \tau_{{{\text{oct}}}} = f\left( {\sigma_{m,2} } \right) $$

where *τ*_*oct*_ and *σ*_*m*,2_ are the octahedral shear stress and the effective intermediate stress, respectively, the expression is:

5 $$ \tau_{oct} = \frac{1}{3}\sqrt {\left( {\sigma_{1} - \sigma_{2} } \right)^{2} + \left( {\sigma_{2} - \sigma_{3} } \right)^{2} + \left( {\sigma_{3} - \sigma_{1} } \right)^{2} } $$

6 $$ \sigma_{m,2} = {{\left( {\sigma_{1} + \sigma_{3} } \right)} \mathord{\left/ {\vphantom {{\left( {\sigma_{1} + \sigma_{3} } \right)} 2}} \right. \kern-\nulldelimiterspace} 2} $$

Since *σ*_2_ = *σ*_3_ in the conventional triaxial test, so:

7 $$ \tau_{oct} = \frac{\sqrt 2 }{3}\left( {\sigma_{1} - \sigma_{3} } \right) $$

Al-Ajmi and Zimmerman^31,32^ found that there is a linear relationship between *τ*_*oct*_ and *σ*_*m*,2_, and proposed the Mogi–Coulomb strength criterion:

8 $$ \tau_{{{\text{oct}}}} = a + b\sigma_{m,2} $$

where *a* and *b* are test parameters.

By comparing Eqs. (6) to (8) with the Mohr–Coulomb expression, the relationship between the test parameters *a*, *b* and the Mohr–Coulomb parameters *c*, *φ* can be obtained:

9 $$ a = \frac{2\sqrt 2 }{3}c\cos \varphi $$

10 $$ b = \frac{2\sqrt 2 }{3}\sin \varphi $$

The Drucker–Prager strength criterion not only considers the influence of the intermediate principal stress, but also considers the effect of hydrostatic pressure, the expression is:

11 $$ \sqrt {J_{2} } = \alpha I_{1} + {\text{k}} $$

where *α* and *k* are experimental constants, *I*_1_ is the first invariant of stress, and *J*_2_ is the second invariant of stress deviator. The expressions of *I*_1_ and *J*_2_ are:

12 $$ I_{1} = \sigma_{1} + \sigma_{2} + \sigma_{3} $$

13 $$ J_{2} = \frac{1}{6}\left[ {\left( {\sigma_{1} - \sigma_{2} } \right)^{2} + \left( {\sigma_{2} - \sigma_{3} } \right)^{2} + \left( {\sigma_{3} - \sigma_{1} } \right)^{2} } \right] $$

The experimental constants in the D–P criterion can be converted to the Mohr–Coulomb parameters in four ways ^33,34^: (1) the M–C outer corner circumscribed circle criterion (DP1), (2) the M–C inner corner circumscribed circle criterion (DP2), (3) the M–C inscribed circle criterion (DP3), (4) M–C equal-area circle criterion (DP4), the specific expressions are shown in Table 2. The correct application of the D–P series of criteria depends on the different stress states of the rock mass. For the stress state that satisfies the conditions of *σ*_1_ > *σ*_2_ = *σ*_3_, such as unidirectional compression and conventional triaxial compression, DP1 matches the M-C criterion, so this paper selects the DP1 criterion for analysis.

**Table 2:** The parameters relationship of D–P criterion and M–C criterion.

Numbering	Type	*α*	*k*
DP1	M–C outer corner circumscribed circle criterion	$\alpha { = }\frac{2\sin \varphi }{{\sqrt 3 \left( {3 - \sin \varphi } \right)}}$	${\text{k = }}\frac{{6{\text{c}}\cos \varphi }}{{\sqrt 3 \left( {3 - \sin \varphi } \right)}}$
DP2	M–C inner corner circumscribed circle criterion	$\alpha { = }\frac{2\sin \varphi }{{\sqrt 3 \left( {3 + \sin \varphi } \right)}}$	${\text{k = }}\frac{{6{\text{c}}\cos \varphi }}{{\sqrt 3 \left( {3 + \sin \varphi } \right)}}$
DP3	M–C inscribed circle criterion	$\alpha { = }\frac{\sin \varphi }{{\sqrt 3 \sqrt {3{ + }\sin^{2} \varphi } }}$	${\text{k = }}\frac{{{\text{3c}}\cos \varphi }}{{\sqrt 3 \sqrt {3{ + }\sin^{2} \varphi } }}$
DP4	M–C equal-area circle criterion	$\alpha { = }\frac{2\sqrt 3 \sin \varphi }{{\sqrt {2\sqrt 3 \pi \left( {9 - \sin^{2} \varphi } \right)} }}$	${\text{k = }}\frac{{6\sqrt 3 {\text{c}}\cos \varphi }}{{\sqrt {2\sqrt 3 \pi \left( {9 - \sin^{2} \varphi } \right)} }}$

**Figure 6:**
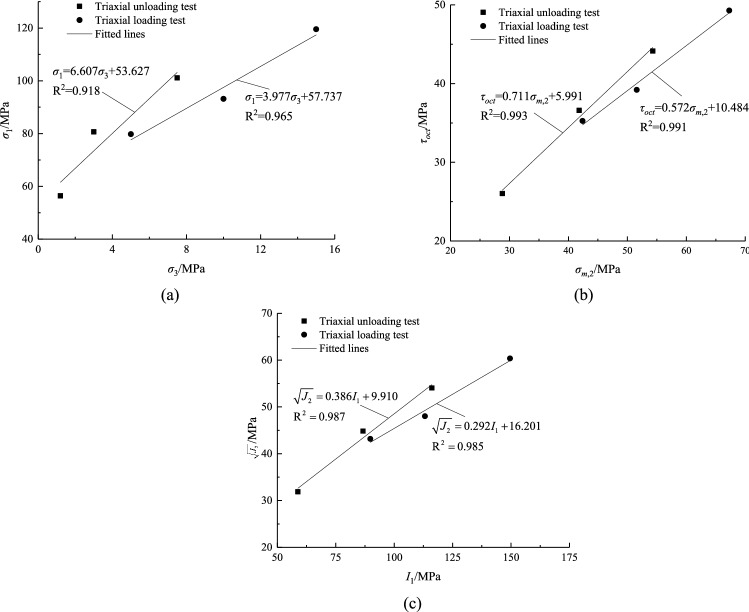
Three kinds of strength criterion regression fitting: (**a**) Mohr–Coulomb, (**b**) Mogi–Coulomb, (**c**) Drucker–Prager.

According to the test data in Table 1, regression analysis was performed using Eqs. (1), (8) and (11), as shown in Fig. 6. The *c* and *φ* are obtained through calculation methods under different criteria, as shown in Table 3. Similarly, the above method is used to solve the residual shear strength parameters *c*_r_ and *φ*_r_, as shown in Table 4.

**Table 3:** *c* and *φ* values of argillaceous sandstone under different criteria.

Strength criterion	Triaxial loading test			Triaxial unloading test		
	*c* (MPa)	*φ* (°)	*f*_*a*_	*c* (MPa)	*φ* (°)	*f*_*a*_
Mohr–Coulomb	14.48	36.74	8.67	10.43	47.48	14.41
Mogi–Coulomb	13.99	37.38	1.60	9.67	48.92	1.78
Drucker–Prager	14.07	37.28	2.47	8.68	48.76	2.91

**Table 4:** *c*_r_ and *φ*_r_ values of argillaceous sandstone under different criteria.

Strength criterion	Triaxial loading test			Triaxial unloading test		
	*c*_r_ (MPa)	*φ*_r_ (°)	*f*_*a*_	*c*_r_ (MPa)	*φ*_r_ (°)	*f*_*a*_
Mohr–Coulomb	8.78	18.22	162.39	4.08	31.97	155.97
Mogi–Coulomb	8.74	18.43	0.38	4.43	30.99	0.93
Drucker–Prager	8.22	18.42	0.53	2.09	21.19	3.70

It can be seen from Fig. 6 that the Mogi–Coulomb and Drucker–Prager strength criterion under different stress paths have a better regression effect than the Mohr–Coulomb. The intermediate principal stress is considered in the first two strength criteria, indicating that it has a certain influence on rock failure. In order to judge which parameter is more accurate, one method is to make preliminary judgments based on the engineering field experience, and the other method is to use mathematical methods to calculate the sum of the absolute values of the fitting deviations for optimization^35^.Therefore, the average strength deviation *f*_*a*_ is introduced:

14 $$ {\text{f}}_{a} { = }\sum {\frac{{ABS\left( {{\text{f}}_{c} - {\text{f}}_{t} } \right)}}{N}} $$

where *f*_*c*_ and *f*_*t*_ are the calculated and experimental values of *σ*_1_, *τ*_*oct*_ and $\sqrt {J_{2} }$ in the three criteria, and *N* is the number of groups of test data.

The final results of the two methods both show that *c,* *φ,* *c*_r_ and *φ*_r_ obtained by the Mogi–Coulomb are more appropriate. The Mogi-Coulomb can better reflect the loading and unloading failure strength and residual strength characteristics of argillaceous sandstone. It can be calculated from Tables 3 and 4 that *c* decreased by 30.87%, *c*_r_ decreased by 49.31%, *φ* increased by 30.87%, and *φ*_r_ increased by 68.15% in the unloaded state compared with the loaded state. Before the rock reaches its peak strength during unloading, the tensile cracks produced will destroy the rock particles and cements, and eventually cause the rock *c* to decrease. The tensile-shear fracture surface is generally rougher than the compression-shear fracture surface, so *φ* will increase when the rock is unloaded.

## Analysis of rock fracture surface characteristics and failure mechanism

### Geometric characteristics of fracture surface

**Figure 7:**
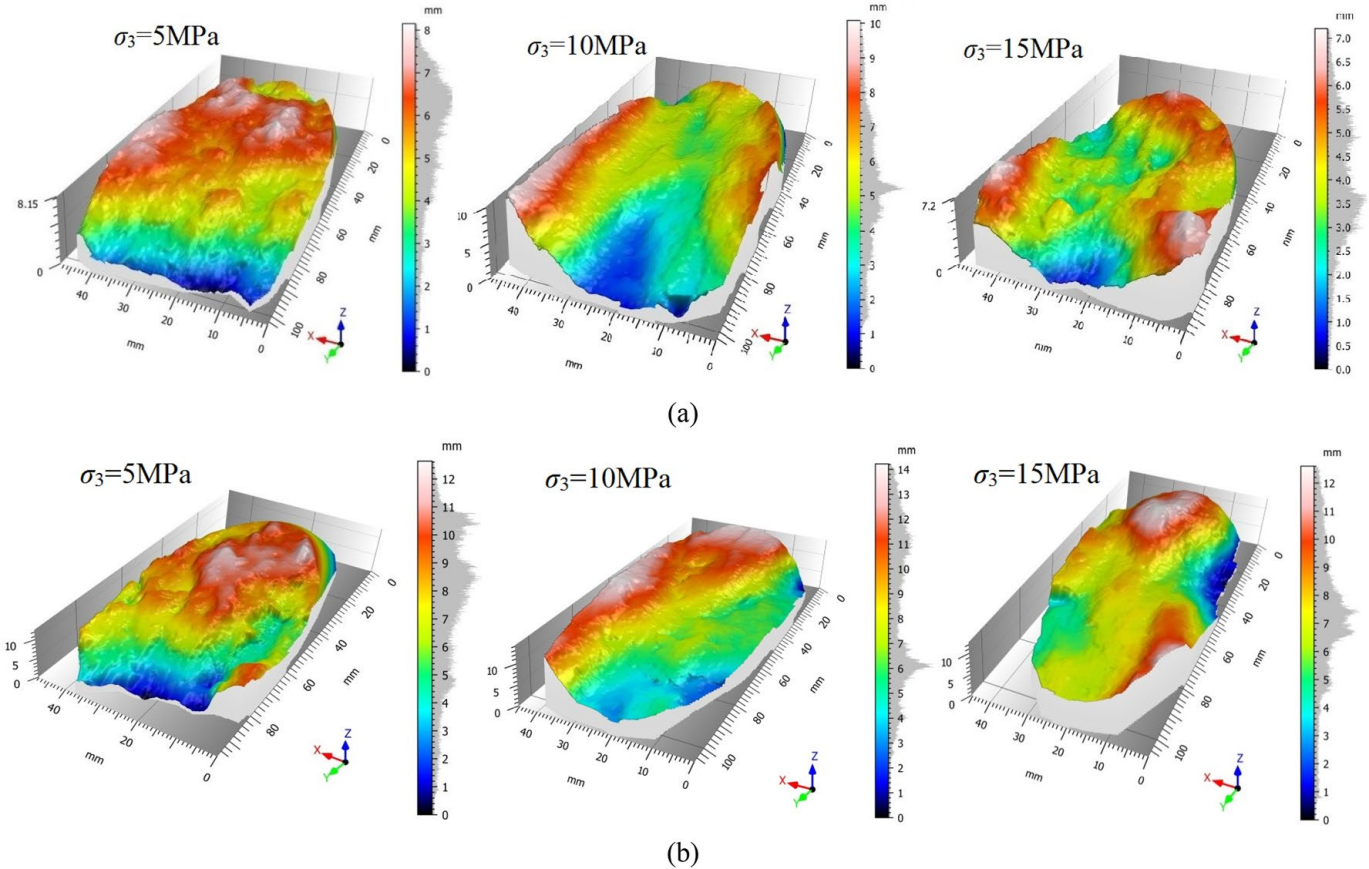
Three-dimensional topography of fracture surface: (**a**) triaxial loading test, (**b**) triaxial unloading test.

In order to analyze the difference in the meso-structure characteristics of the rock fracture surface, the ST500 three-dimensional profile scanner was used to scan the fracture section of argillaceous sandstone, as shown in Fig. 7. Taking the plane with the smallest sum of squares of the distance from each point in the sampling area to the plane as the reference plane, six height characteristic parameters and two texture parameters are selected to reflect the characteristics of the fracture surface's roughness, dispersion and skewness^36^. The height characteristic parameters include the maximum surface peak height *S*_*p*_, the maximum valley depth on the surface *S*_*m*_, the maximum profile height *S*_*h*_, the arithmetic mean deviation *S*_*a*_, the height root mean square *S*_*q*_ and the kurtosis coefficient *Sku*. Texture parameters include autocorrelation length *Sal* and texture aspect ratio *Str*. The morphological characteristics and calculation formulas reflected by each parameter are shown in Table 5.

**Table 5:** Three-dimensional profile characteristic parameters.

Parameters	Reflected morphology characteristics and calculation formula
Maximum surface peak height *S*_*p*_	The distance from the highest point in the sampling area to the reference plane:$S_{{\text{p}}} = \max (S_{p1} ,S_{p2} , \cdot \cdot \cdot ,S_{pn} )$。
Maximum valley depth on the surface *S*_*m*_	The distance from the lowest point in the sampling area to the reference plane:$S_{m} = \max (S_{m1} ,S_{m2} , \cdot \cdot \cdot ,S_{mn} )$。
Maximum profile height *S*_*h*_	It is the sum of *S*_*p*_ and *S*_*m*_: $S_{h} = S_{p} + S_{m}$, which can better reflect the height of the largest peak and valley of the section
Arithmetic mean deviation *S*_*a*_	The arithmetic mean of the distance from each point on the surface to the reference plane: $S_{a} = \frac{1}{A}\iint\limits_{{D_{xy} }} {\left| {z(x,y)} \right|}dxdy$. It is an important parameter reflecting the surface roughness
Height root mean square *S*_*q*_	The square root of the sum of the squares of the distances from each point on the surface to the reference plane: $S_{q} = \frac{1}{A}\sqrt {\iint\limits_{{D_{xy} }} {z^{2} (x,y)dxdy}}$, which can better reflect the discrete and volatility of the surface topography
Kurtosis coefficient *Sku*	It is used to quantitatively describe the flatness and concentration of the surface height distribution. If *Sku* = 3, it means that the section surface height distribution is a normal distribution. *Sku* < 3 and *Sku* > 3 respectively indicate that the height distribution is relatively scattered and relatively concentrated
Autocorrelation length *Sal*	The autocorrelation length is the length that decays to 0.2 fastest. The larger the *Sal*, the higher the correlation between points on the surface, that is, the gentle changes in surface height have major low-order undulations
Texture aspect ratio *Str*	Indicates the degree of homogeneity and anisotropy on the surface. The value of *Str* is 0 ~ 1. If *Str* > 0.5 and *Str* < 0.3, it indicates that the surface has strong allotropic and anisotropic features, respectively

*S*_*pi*_ and *S*_*mj*_ (i = 1, 2,…, n) in the table are the distances from all contour peaks and contour peak valleys to the reference plane respectively, and *A* is the area of the sampling area.

The three-dimensional morphological parameters of argillaceous sandstone fractured surface, as shown in Table 6. The three-dimensional topography parameters of the fracture surface of argillaceous sandstone change regularly with the increase of *σ*_3_, which indicates that the characteristics of the fracture surface are related to the *σ*_3_. The fracture parameters *S*_*p*_, *S*_*m*_, *S*_*h*_, *S*_*a*_ and *S*_*q*_ of argillaceous sandstone first increase and then decrease with the increase of *σ*_3_ under triaxial loading. When *σ*_3_ = 10 MPa, the undulation, volatility and roughness of the fractured surface are the largest, followed by *σ*_3_ = 5 MPa, and *σ*_3_ = 15 MPa is the smallest. When the *σ*_3_ = 5 MPa, the rock sample exhibits brittle failure and the fracture surface morphology is more complicated because the confining pressure has less restriction on lateral deformation. However, the fracture surface will continue to be worn after the rock sample is destroyed, resulting in a decrease in the undulation, volatility and roughness of the final fracture surface. When *σ*_3_ is increased to 15 MPa, the lateral deformation of the rock sample can be better restrained. The internal particle friction of the rock sample is more serious and exhibits strong plasticity, so that the fractured section is relatively smooth. When *σ*_3_ = 10 MPa, the fracture surface at failure is more complex than that at 15 MPa, and the fracture surface wears less after failure than at 5 MPa, resulting in more complex characteristics of the final fracture surface height. The kurtosis coefficient *Sku* is greater than 3 when *σ*_3_ = 5 MPa, indicating that the height distribution of the fracture surface is more concentrated, and the height distribution under other confining pressures is more scattered. The autocorrelation length Sal is the largest when *σ*_3_ = 5 MPa, that is, the change of the fracture surface height is the most gentle.

**Table 6:** Three-dimensional morphological parameters of argillaceous sandstone fractured surface.

Test type	*σ*_3_ (MPa)	*S*_*p*_ (mm)	*S*_*v*_ (mm)	*S*_*h*_ (mm)	*S*_*a*_ (mm)	*S*_*q*_ (mm)	*Sku*	*Sal* (μm)	*Str*
Triaxial loading test	5	2.76	5.40	8.15	1.18	1.48	3.47	18.70	0.66
	10	4.92	5.17	10.10	1.35	1.74	2.89	10.40	0.42
	15	3.24	3.95	7.20	1.04	1.28	2.85	12.20	0.72
Triaxial unloading test	5	5.02	7.62	12.60	1.98	2.44	2.78	16.30	0.82
	10	5.88	8.33	14.20	2.65	2.99	1.85	9.19	0.17
	15	7.89	7.97	15.80	2.90	3.58	2.40	8.64	0.62

Under the condition of unloading, the height characteristic parameters *S*_*p*_, *S*_*m*_, *S*_*h*_, *S*_*a*_ and *S*_*q*_ increase with the increase of confining pressure or peak strength. It shows that the higher the stress level when the argillaceous sandstone is unloaded, the greater the undulation, volatility and roughness of the fracture surface after failure. The kurtosis coefficient *Sku* is all less than 3, and the height distribution of the fracture surface is relatively scattered. When *σ*_3_ = 15 MPa, the autocorrelation length *Sal* is the smallest, and the height of the fracture surface fluctuates the most.

Under the same confining pressure, the height characteristic parameters *S*_*p*_, *S*_*m*_, *S*_*h*_, *S*_*a*_ and *S*_*q*_ of fracture surface are larger when unloading than when loading. That is, the undulation, volatility and roughness of the fracture surface are greater when unloading. The *Sal* under the triaxial loading condition is larger, and the fracture surface height changes relatively smoothly, but the opposite under the unloading condition. The texture aspect ratio *Str* of the fracture surface is greater than 0.5 when the *σ*_3_ = 5 MPa and *σ*_3_ = 15 MPa, indicating that the fracture surface has strong isotropic characteristics. In general, the morphological parameters of the fracture surface of argillaceous sandstone under triaxial compression and unloading conditions are quite different. This is related to the development of microcracks, that is, the failure mechanism of the argillaceous sandstone under the two stress paths is different.

### Failure process and mechanism of argillaceous sandstone

**Figure 8:**
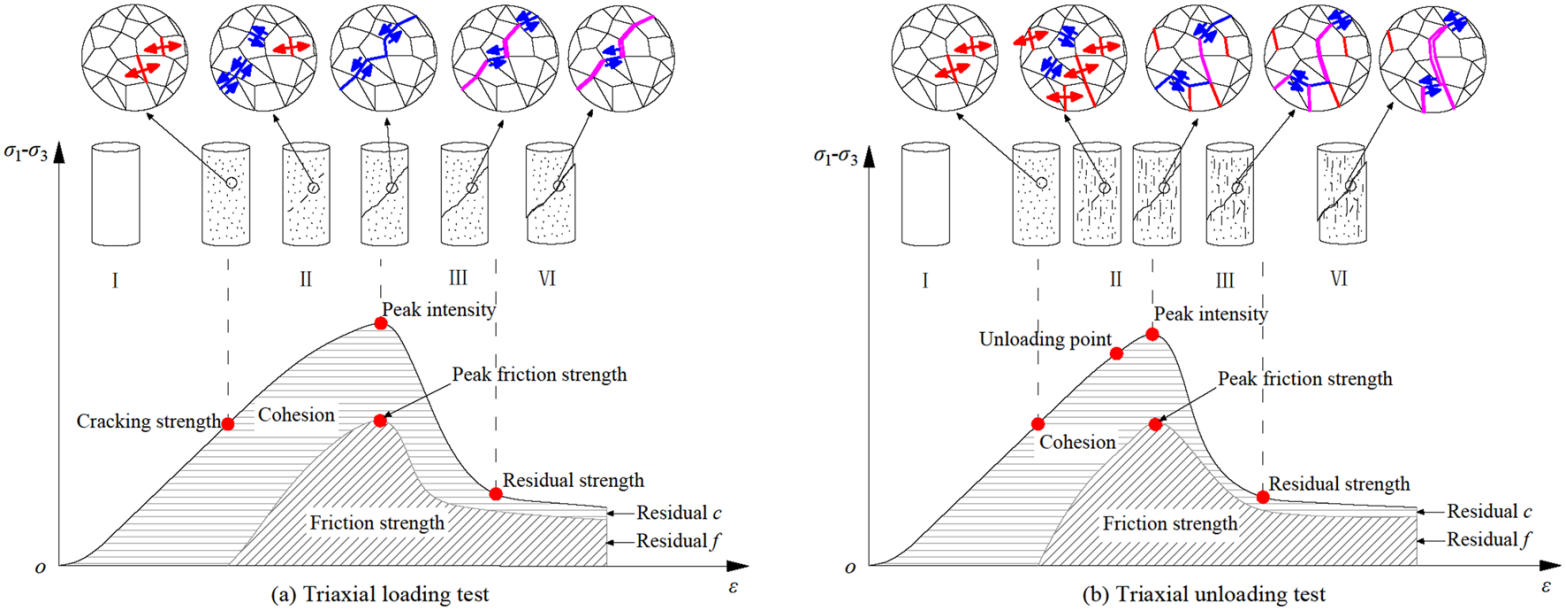
Schematic diagram of rock failure process under different stress paths.

Based on the stress–strain curves, shear strength parameters, failure characteristics, and geometric characteristics of the fracture surface of argillaceous sandstone, the failure process is described, as shown in Fig. 8. Combining the dynamic CWFS (Cohesive Weakening and Frictional Strengthening) model^37^ of cohesion ($f(c,\overline{\varepsilon }^{p} )$), friction strength ($f(\sigma_{n} ,\overline{\varepsilon }^{p} )\tan \varphi$) and plastic strain ($\overline{\varepsilon }^{p}$), the effect of strength parameters on the deformation and failure process of rock is analyzed. Where, the cohesion and friction strength are functions related to the effective plastic strain, and the effective plastic strain can be obtained by the rock triaxial compressive stress strain curve. And then, based on the development of micro-fractures, the failure process can be described in four stages: the development stage without micro-cracks (I), the development and penetration stage of micro-cracks (II), the macro-fracture surface formation stage (III), and the complete fracture stage (IV).

The development stage without micro-cracks (I): The axial force does not exceed the rock’s cracking strength. At this stage, the rock’s own micro-cracks are compacted and closed, and no new micro-cracks appear in the rock particles and cements. At this time, the axial force is mainly borne by the cohesive force between the rock particles, so the cohesive is the maximum when a new crack appears, and the friction strength is approximately zero.

The development and penetration stage of micro-cracks (II): when the axial stress exceeds the cracking strength, the rock begins to break and form new micro-cracks. With the increase of the axial stress, the micro-cracks gradually converge and penetrate. As the connection between particles breaks to form shear cracks or tension cracks, the cohesive of the rock gradually weakens, and the friction force begins to take effect. In the triaxial unloading test, confining pressure unloading will be carried out at this stage, and the reduction of lateral restraint will result in the formation of tension cracks perpendicular to the unloading direction. The connection force between the particles and the friction between the shear cracks will decrease, resulting in a lower load-bearing capacity of the rock than when loaded. At the same time, due to the influence of axial pressure and confining pressure, the development of micro-cracks during loading is mainly the penetration of shear cracks. In the unloaded state, some of the rock bridges between the tensile cracks will be sheared, thereby forming a tensile-shear or shear-tension through fracture zone. Therefore, compared with loading, the fracture surface of the rock in the unloaded state is more complicated, with greater roughness, volatility and undulation, lower cohesion, and greater friction angle.

The macro-fracture surface formation stage (III): when the load exceeds the peak strength, the bearing capacity of the rock begins to decrease. Micro-cracks develop into macro-cracks, gradually forming a macro-fracture surface. At this stage, part of the rock particles on the shear surface that block the sliding of the shear surface will be sheared or worn, resulting in a further reduction in cohesion and friction strength.

The complete fracture stage (IV): in the residual strength stage of the stress–strain curve, the macroscopic fracture surface has been completely formed, and the argillaceous sandstone bearing capacity is basically unchanged. The residual cohesion and the residual friction strength are basically stable, and the contribution of the residual cohesion in the residual strength is small.

## Conclusions

1. The argillaceous sandstone is mainly damaged by compression shear when triaxially loaded. Since the unloading of the confining pressure will form a tensile-shear crack perpendicular to the unloading direction, the tensile-shear failure is the main damage during triaxial unloading. The stress path has a great influence on the *c* and *φ* values of argillaceous sandstone. Compared to the loaded condition, *c* decreased by 30.87% and *φ* increased by 30.87% under the unloaded condition. For argillaceous sandstone in this paper, the Mogi–Coulomb can better reflect the strength characteristics of argillaceous sandstone than the Mohr–Coulomb and Drucker–Prager.
2. From the six height characteristic parameters and two texture parameters of the fracture surface topography, it can be seen that the undulation, fluctuation and roughness of the fracture surface are larger during unloading than during loading. It shows that the microfracture development process of argillaceous sandstone is different under the two stress paths.
3. Based on the development of rock micro-cracks, the damage process mainly has the following four stages: the development stage without micro-cracks, the development and penetration stage of micro-cracks, the macro-fracture surface formation stage and the complete fracture stage. During the unloading stage of micro-crack development and penetration, tension cracks will be formed, resulting in tension shear failure. The occurrence of tension cracks is the main reason for the differences in mechanical parameters, failure characteristics and fracture surface morphology characteristics of argillaceous sandstone under loading and unloading.

## Data Availability

The datasets used and/or analysed during the current study available from the corresponding author on reasonable request.

## References

[1] Zhang H, Chen L, Chen SG, Sun JC, Yang JS (2018). The spatiotemporal distribution law of microseismic events and rockburst characteristics of the deeply buried tunnel group. Energies.

[2] Yu WJ, Pan B, Zhang F, Yao SF, Liu FF (2019). Deformation characteristics and determination of optimum supporting time of alteration rock mass in deep mine. KSCE J. Civ. Eng..

[3] Liang YP, Li QM, Gu YL, Zou QL (2017). Mechanical and acoustic emission characteristics of rock: Effect of loading and unloading confining pressure at the postpeak stage. J. Nat. Gas Sci. Eng..

[4] Zhao HG, Song ZL, Zhang DM, Liu C, Yu BC (2021). True triaxial experimental study on mechanical characteristics and energy evolution of sandstone under various loading and unloading rates. Geomech. Geophys. Geo..

[5] Gao F, Zhou KP, Luo XW, Zhai JB (2012). Effect of induction unloading on weakening of rock mechanics properties. Trans. Nonferr. Metal Soc..

[6] Ding QL, Ju F, Mao XB, Ma D, Yu BY, Song SB (2016). Experimental investigation of the mechanical behavior in unloading conditions of sandstone after high-temperature treatment. Rock Mech. Rock Eng..

[7] Liu QQ, Cheng YP, Jin K, Tu QY, Zhao W, Zhang R (2017). Effect of confining pressure unloading on strength reduction of soft coal in borehole stability analysis. Environ. Earth Sci..

[8] Wang Y, Qiao QN, Li JL (2019). The effect of initial creep damage on unloading failure properties of sandstone from macro-mesoscopic perspective. Period Polytech-Civ..

[9] Xu H, Feng XT, Yang CX, Zhang XW, Zhou YY, Wang ZF (2019). Influence of initial stresses and unloading rates on the deformation and failure mechanism of Jinping marble under true triaxial compression. Int. J. Rock Mech. Min. Sci..

[10] Meng QB, Liu JF, Xie LX, Pu H, Yang YG, Huang BX, Qian W (2022). Experimental mechanical strength and deformation characteristics of deep damaged-fractured rock. B Eng. Geol. Environ..

[11] Xin CP, Wang K, Du F, Zhang X, Wang GD, Liu YL (2018). Mechanical properties and permeability evolution of gas-bearing coal under phased variable speed loading and unloading. Arab. J. Geosci..

[12] Duan K, Ji YL, Wu W, Kwok CY (2019). Unloading-induced failure of brittle rock and implications for excavation-induced strain burst. Tunn. Undergr. Sp. Tech..

[13] Yang YS, Zhang DM, Zhang BG, Ye C, Yang BY (2019). Analysis of Strength and permeability of crystalline sandstone under loading-unloading conditions. Energ. Source Part A..

[14] Wu FQ, Liu T, Liu JY, Tang XL (2009). Excavation unloading destruction phenomena in rock dam foundations. B Eng. Geol. Environ..

[15] Yang SQ, Jing HW, Wang SY (2012). Experimental investigation on the strength, deformability, failure behavior and acoustic emission locations of red sandstone under triaxial compression. Rock Mech. Rock Eng..

[16] Lu YH, Liu QS, Jiang H (2010). Study of mechanical deformation characteristics of granite in unloading experiments of high stress. Rock Soil Mech..

[17] Li DY, Sun Z, Xie T, Li XB, Ranjith PG (2017). Energy evolution characteristics of hard rock during triaxial failure with different loading and unloading paths. Eng. Geol..

[18] Qiu SL, Feng XT, Xiao JQ, Zhang CQ (2013). An experimental study on the pre-peak unloading damage evolution of marble. Rock Mech. Rock Eng..

[19] Cong Y, Wang ZQ, Zheng YR, Zhang LM (2020). Effect of unloading stress levels on macro- and microfracture mechanisms in brittle rocks. Int. J. Geomech..

[20] Zhou L, Gao WT, Yu LY, Zhu ZM, Chen JX, Wang XK (2022). Thermal effects on fracture toughness of cracked straight-through Brazilian disk green sandstone and granite. J. Rock Mech. Geotech..

[21] Zhou L, Zhu ZM, Liu RF, Fan Y, Dong YQ, Ying P (2020). Investigation on fracture properties of single-flawed tunnel model under medium-to-low-speed impacts. Acta Mech. Solida Sin..

[22] Zhao XG, Wang J, Cai M, Cheng C, Ma LK, Su R, Zhao F, Li DJ (2013). Influence of unloading rate on the strainburst characteristics of beishan granite under true-triaxial unloading conditions. Rock Mech. Rock Eng..

[23] Zhou XP, Zhang YX, Ha QL (2008). Real-time computerized tomography (CT) experiments on limestone damage evolution during unloading. Theor. Appl. Fract. Mech..

[24] Nasseri MHB, Grasselli G, Mohanty B (2009). Fracture toughness and fracture roughness in anisotropic granitic rocks. Rock Mech. Rock Eng..

[25] Ban LR, Qi CZ, Chen HX, Yan FY, Ji CM (2019). A new criterion for peak shear strength of rock joints with a 3D roughness parameter. Rock Mech. Rock Eng..

[26] Yang SQ, Yin PF, Huang YH, Cheng JL (2019). Strength, deformability and X-ray micro-CT observations of transversely isotropic composite rock under different confining pressures. Eng. Fract. Mech..

[27] Wang ZH, Yang SL, Tang YS (2020). Mechanical behavior of different sedimentary rocks in the Brazilian test. B Eng. Geol. Environ..

[28] Fairhurst CE, Hudson JA (1999). Draft ISRM suggested method for the complete stress–strain curve for intact rock in uniaxial compression. Int. J. Rock Mech. Min. Sci..

[29] Yazdani Bejarbaneh B, JahedArmaghani D, Mohd Amin MF (2015). Strength characterisation of shale using Mohr–Coulomb and Hoek–Brown criteria. Measurement.

[30] Mogi, K. *Experimental**Rock**Mechanics*. 50–58. (Taylor & Francis Group, 2007).

[31] Al-Ajmi AM, Zimmerman RW (2005). Relation between the Mogi and the Coulomb failure criteria. Int. J. Rock Mech. Min. Sci..

[32] Al-Ajmi AM, Zimmerman RW (2005). Stability analysis of vertical boreholes using the Mogi–Coulomb failure criterion. Int. J. Rock Mech. Min. Sci..

[33] Zhang LY, Cao P, Radha KC (2010). Evaluation of rock strength criteria for wellbore stability analysis. Int. J. Rock Mech. Min. Sci..

[34] Deng CJ, He GJ, Zheng YR (2006). Studies on Drucker–Prager yield criterions based on M–C yield criterion and application in geotechnical engineering. Chin. J. Geotech. Eng..

[35] You MQ (2009). True-triaxial strength criteria for rock. Int. J. Rock Mech. Min. Sci..

[36] Wang Y, Ai Q, Li JL, Deng HF (2019). Damage characteristics of sandstone under different influence factors and its unloading failure meso-morphology properties. Rock Soil Mech..

[37] Hajiabdolmajid V, Kaiser P (2003). Brittleness of rock and stability assessment in hard rock tunneling. Tunn. Undergr. Sp. Tech..

